# NLRP1 Overexpression Is Correlated with the Tumorigenesis and Proliferation of Human Breast Tumor

**DOI:** 10.1155/2017/4938473

**Published:** 2017-10-29

**Authors:** Yuxian Wei, Hongyan Huang, Zhu Qiu, Hongzhong Li, Jinxiang Tan, Guosheng Ren, Xiaoyi Wang

**Affiliations:** ^1^Department of Breast and Endocrine Surgery, The First Affiliated Hospital of Chongqing Medical University, Chongqing 400016, China; ^2^Chongqing Key Laboratory of Molecular Oncology and Epigenetics, The First Affiliated Hospital of Chongqing Medical University, Chongqing 400016, China

## Abstract

Recent studies suggest that nucleotide-binding domain leucine-rich repeat protein 1 (NLRP1) is a pivotal factor in the inflammatory process. However, the role of NLRP1 in breast cancer pathogenesis remains unclear. The aim of this study was to examine the expression and function of NLRP1 in breast cancer. We found that NLRP1 was widely expressed in 83% (60/72) of primary breast cancer tissue. NLRP1 expression level was higher in primary breast cancer tissue than in adjacent noncancerous tissue (*p* < 0.001) and NLRP1 expression was associated with lymph node metastasis (*p* = 0.003), TNM stage (*p* = 0.003), and Ki-67 levels (*p* < 0.001). Overexpression of NLRP1 in the breast cancer cell line MCF-7 promotes proliferation, migration, invasion, and tumorigenicity in nude mice. Restoration of NLRP1 expression resulted in the EMT occurrence that downregulation of epithelial marker E-cadherin and upregulation mesenchymal marker vimentin, C-myc, MMP-9, and snail. In summary, NLRP1 promotes cell line MCF-7 the proliferation, migration, and invasion through inducing EMT.

## 1. Introduction

Breast cancer is a leading cause of cancer related death in women [1, 2]. Inflammation has been related to breast cancer incidence and progression with nucleotide-binding domain leucine-rich repeat proteins (NLRs) thought to play a potential inflammatory role in not only breast cancer but also a variety of other disease processes [3, 4]. NLRP1 (also known as CARD7, NAC, DEFCAP, and NALP1) was the first identified NLR-family protein [5, 6] and differs from other NLR-family proteins in that its C-terminal region contains a nucleotide-binding domain (NBD). It is similar to other family members in that it contains conserved leucine-rich repeat (LRR) domains [7, 8]. NLRP1 was the first protein demonstrated to form an inflammasome with the capacity to activate caspases and to induce a cellular inflammatory response [6, 9–15]. Some authors demonstrated that NLRP1 promotes melanoma growth by enhancing inflammasome activation and suppressing apoptotic pathways [16]. Inversely, other authors demonstrated that NLRP1 attenuates colitis and colitis-associated tumorigenesis [17]. But the expression and functional role of NLRP1 in primary breast cancer has not been reported previously.

In this study, we overexpressed NLRP1 in human breast cancer MCF-7 cell and established xenograft tumor nude mice model and then observed that this protein promotes breast tumor migration, invasion, and growth.

## 2. Materials and Methods

### 2.1. Tissue Specimens and Immunohistochemistry

Primary breast carcinomas and corresponding tumor-margin tissues were obtained from patients at the First Affiliated Hospital of Chongqing Medical University (Chongqing, China). All samples were collected with informed consent from patients, and all experimental procedures were authorized by the Ethics Committee of The First Affiliated Hospital of Chongqing Medical University. Immunohistochemistry was performed using a PV-9000 two-step detection kit (ZSGB-BIO, Beijing, China) as described previously [18]. Thin sections were incubated with mouse anti-human NLRP1 monoclonal antibody (1 : 50 dilution; ab16091; Abcam, Cambridge, MA, USA) at 4°C overnight. Phosphate buffered saline (PBS) served as a negative control. We used the widely accepted German semiquantitative scoring system [19], to evaluate the staining intensity and area extent of stained cells. According to the cells' staining intensity and the area of stained cells, each section was defined a score standard (no staining = 0; weak staining = 1; moderate staining = 2; strong staining = 3) and (0% = 0; 1–24% = 1; 25–49% = 2; 50–74% = 3; 75–100% = 4). The final score was determined by multiplying the cells' staining intensity with the area of stained cells, ranging from 0 (the minimum grad) to 12 (the maximum grad). In our study, we assigned 0–8 score was defined as low expression group, and another group containing 9–12 score was defined as high expression group.

### 2.2. Animal Experiments

Female BALB/c nude mice (4–6 weeks old, *n* = 4, weighing 18–22 g) were provided by the Experimental Animal Center of Chongqing Medical University (CQMU), China. All mice were fed according to institutional and CQMU guidelines for the use of animals. Stable NLRP1-expressing MCF-7 cells or MCF-7 cells transfected with vector alone (1 × 10^7^ cells in 150 *μ*l PBS) were subcutaneously injected, respectively, into the right and separately into the left dorsal flank of each mouse. After 1 week, tumors were visible and tumor lengths and widths were measured by caliper every 3 days. Tumor volumes were calculated using the formula (length × width^2^)/2 [20]. The mice were sacrificed 28 days after inoculation. The tumors were weighed and fixed in 4% paraformaldehyde. Thin sections were obtained for immunohistochemical analysis. The protocol for this* in vivo* tumor model was approved through the Institute Ethics Committee of the First Affiliated Hospital of Chongqing Medical University.

### 2.3. Cell Culture and Transfection

The human MCF-7 cell line was cultured at 37°C with 5% CO_2_ in RPMI 1640 (Gibco-BRL, Eggestein, Germany), supplemented with 10% fetal bovine serum (FBS) (Gibco-BRL) and 100 U/ml penicillin and streptomycin (Gibco-BRL). The transfection procedure was performed as previously described [21]. The NLRP1 plasmid was purchased from GeneChem (Shanghai, China). The constructs were transfected into 1 × 10^6^ cells at a concentration of 4 *μ*g with serum-free RPMI 1640; after 6–8 hours, the serum-free RPMI 1640 was changed to 10% FBS RPMI 1640 and selected by neomycin 48 hours after transfection.

### 2.4. Proliferation Assay

Cell proliferation was measured using Edu kits (RIBOBIO, Guangzhou, China), according to the manufacturer's instructions [22]. Prepared cells were exposed to 25 *μ*M 5-ethynyl-2′-deoxyuridine for 2 hours at 37°C.

### 2.5. Western Blot

Transfected cells were lysed with RIPA lysate buffer (Beyotime Institute of Biotechnology, Jiangsu, China) and protein lysates (40 *μ*g) separated by using sodium dodecylsulfate/polyacrylamide gel electrophoresis (SDS-PAGE) and then transferred to polyvinylidene fluoride (PVDF) membranes. Membranes were incubated overnight at 4°C with primary antibodies reactive with NLRP1 and GAPDH (1 : 1000, Abcam) and then incubated with relevant secondary antibodies (1 : 2000, Cell Signaling Technology, Beverly, MA, USA) for 1 hour at room temperature and visualized using an enhanced chemiluminescence kit (ECL; Amersham Pharmacia Biotech, San Francisco, CA, USA). Blots were developed using a Fujifilm Las-4000 Imaging System (Fujifilm, Tokyo, Japan).

### 2.6. Transwell Assay

Cell migration and invasion were assessed using transwell chambers (8 *μ*m pore size; Corning, Corning, NY, USA) as previously described [23]. Briefly, MCF-7 cells stably expressing NLRP1 or vector were collected and washed twice in serum-free medium. Next, 5 × 10^4^ cells/well were resuspended in 100 *μ*l of serum-free RPMI 1640 and plated onto uncoated 8 *μ*m transwell filter inserts in 24-well plates. The lower chamber contained 600 *μ*l of medium containing 10% FBS as a chemoattractant. The cells were incubated at 37°C in a 5% CO_2_ incubator for 36 hours. The upper chamber was fixed with 4% paraformaldehyde for 15 minutes and then stained with 0.1% crystal violet for 10 minutes. Nonmigratory cells in the upper chamber were removed with a cotton swab. Migratory cells were photographed and the cells in three fields counted with a microscope. To assess the capacity for invasion, relevant cells were plated on Matrigel™-coated transwell filters (Matrigel: serum-free medium = 1 : 8, 60 *μ*l/chamber). Next, 1 × 10^5^ cells/well were resuspended in 100 *μ*l serum-free medium and plated onto Matrigel-coated 8-*μ*m transwell filter inserts in 24-well plates, then incubated for 36 hours. At the end of the culture period, invaded cells on the bottom of the membrane were fixed with cotton swab, stained with crystal violet, and counted. All experiments were independently repeated three times.

### 2.7. Statistical Analyses

All statistical analyses were done using SPSS (IBM company) version 19.0 software. Student's *t*-test was used to analyze the difference in NLRP1 expression between breast carcinoma and adjacent tissues. The associations among NLRP1 and clinic-pathologic parameters were assessed by *χ*^2^ and Fisher's Exact test. Differences were considered statistically significant when *p* values were < 0.05. Significant differences were noted as ^*∗*^*p* < 0.05, ^*∗∗*^*p* < 0.01, and ^*∗∗∗*^*p* < 0.001.

## 3. Results

### 3.1. NLRP1 Expression in Breast Tumor Tissues and Its Association with Clinical Features

Expression of NLRP1 was high in 83% (60/72 specimens) of examined breast tumors. High expression was detected in only 28% (10/36) of adjacent, nontumor tissue (*p* < 0.001) (Table 1).

NLRP1 expression and clinic-pathological features of breast cancer patients were analyzed including age, tumor size, lymph node metastasis, histological grade, TNM stage, estrogen receptor (ER) status, progesterone receptor (PR) status, HER2 status, P53, and Ki-67. Chi-squared analysis showed NLRP1 expression to be associated with lymph node metastasis (*p* = 0.003), TNM stage (*p* = 0.003), and Ki-67 detection (*p* < 0.001) (Table 2).

**Figure 1:**
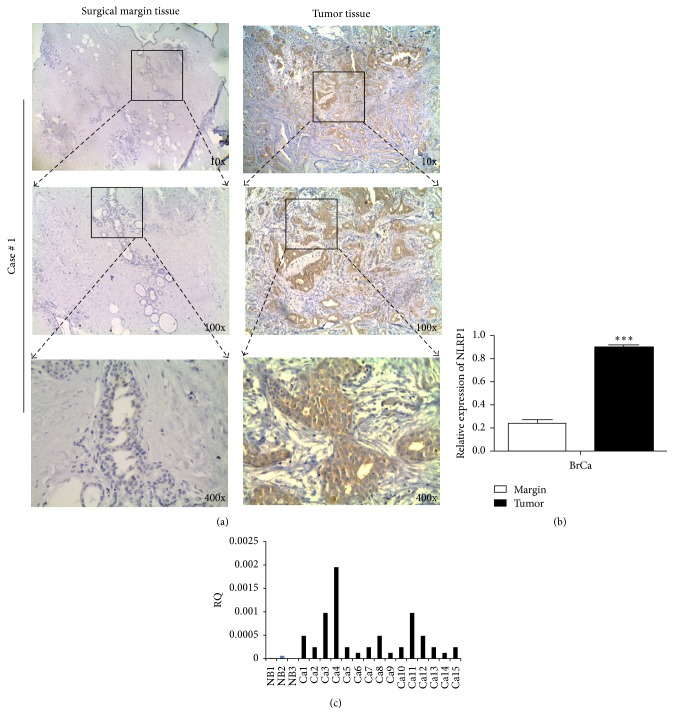
NLRP1 was upregulated in primary breast tumors. (a) Representative immunohistochemical (IHC) staining for NLRP1 in paired breast surgical margin tissues and tumors. Original magnification, 10x, 100x, and 400x. (b) Quantitative analysis: mean optical density (MOD) of NLRP1 expression in paired breast surgical margin tissues and tumors are shown as mean ± SD (^*∗∗∗*^*p* < 0.001, Student's *t*-test). (c) Expression of NLRP1 in human normal and breast tumor tissues detected by qRT-PCR.

Further, the protein and mRNA level of NLRP1 were examined in breast cancer tissue and in adjacent, nontumor tissue by immunohistochemistry and qRT-PCR, as shown in Figure 1(a). The protein level of NLRP1 was significantly increased in breast cancer tissue relative to adjacent, nontumor tissue (Figure 1(b)  *p* < 0.001). The average level of NLRP1 mRNA expression in breast cancer tissues was higher than that in normal breast tissues (Figure 1(c)  *p* < 0.05).

### 3.2. NLRP1 Promotes Breast Cancer Cell Line MCF-7 Proliferation, Migration, and Invasion

**Figure 2:**
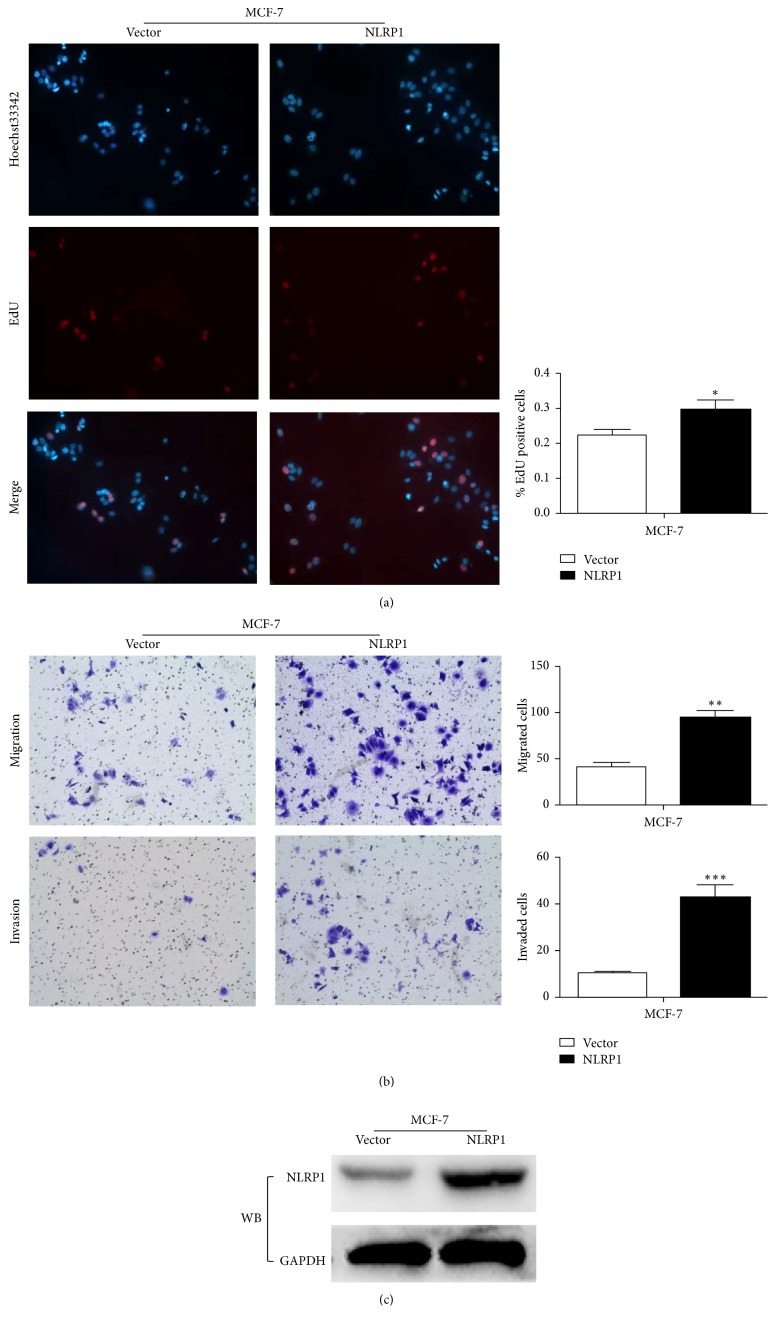
(a) NLRP1 promoted MCF-7 cell proliferation* in vitro *compared with vector by EdU assay (^*∗*^*p* < 0.05). (b) NLRP1 promoted MCF-7 cell migration and invasion* in vitro* compared with vector by transwell assay, representative images of migration, and invasion; the pictures were taken at 36 h after seeding (magnification, ×200) (^*∗∗*^*p* < 0.01, ^*∗∗∗*^*p* < 0.001). (c) Overexpression of NLRP1 in MCF-7 cell was confirmed by western blot.

In the breast cancer cell line MCF-7. Proliferation of NLRP1 stably transfected MCF-7 cells was greater than wild-type, nontransformed MCF-7 cells (as judged by the EdU assay) (Figure 2(a)  *p* < 0.05). We also investigate the effect of NLRP1 on MCF-7 cell migration and invasion and found that the number of migrated and invaded cells was significantly increased in cells transfected with NLRP1, when compared to nontransfected MCF-7 cells (Figure 2(b)). These results demonstrate that NLRP1 transfection promotes the proliferation, migration, and invasion of the breast cancer cell line MCF-7. Overexpression of NLRP1 was confirmed by western blot analysis (Figure 2(c)).

### 3.3. NLRP1 Induces Epithelial-Mesenchymal Transition

**Figure 3:**
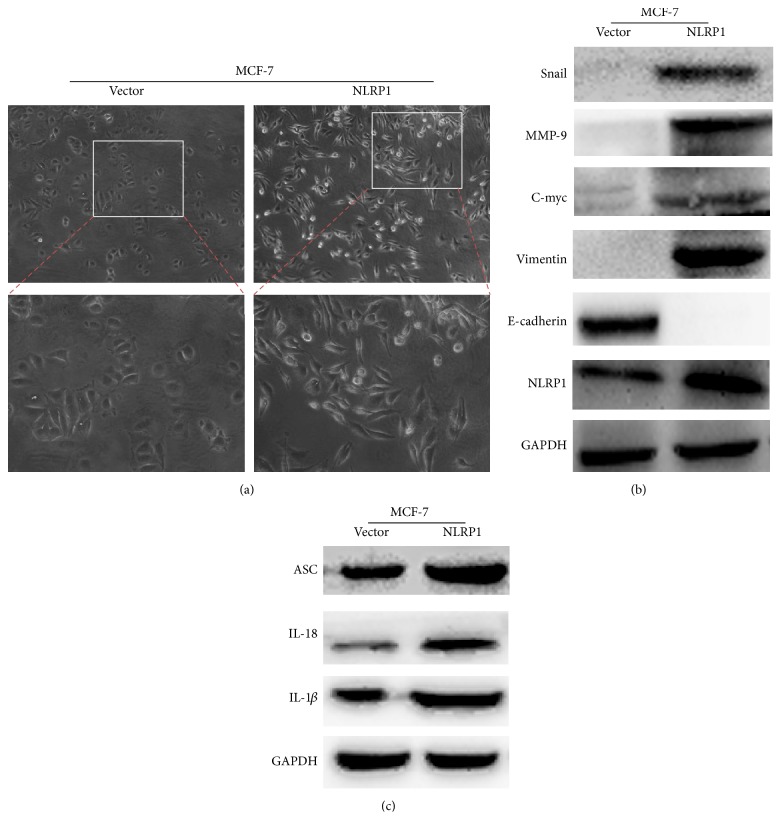
(a) Morphology changes of MCF-7 cells transfected with NLRP1 or vector by phase-contrast microscopy. ((b) and (c)) Western blot analysis of EMT and inflammasome associated markers. GAPDH was used as an internal control.

To assess the effect of NLRP1 on MCF-7 cell migration, we firstly examined cell morphology changes. The vector transfected cells kept cell-cell contacts and adherence to each other, with less aggressive behavior. Whereas, NLRP1-transfected cells exhibited unique irregular shapes and distinguishing spread features (Figure 2), suggesting that NLRP1 more likely induced tumor epithelial-mesenchymal transition (EMT). Associated markers about EMT and inflammasomes were detected by western blot; the results showed that upregulated mesenchymal marker vimentin, C-myc, MMP-9, Snail, IL-1*β*, IL-18, and adaptor protein ASC (apoptosis-associated speck-like protein), then, downregulated epithelial marker E-cadherin in NLRP1-expressing cells (Figures 3(a) and 3(b)).

### 3.4. NLRP1 Promotes Breast Tumor Growth* In Vivo*

**Figure 4:**
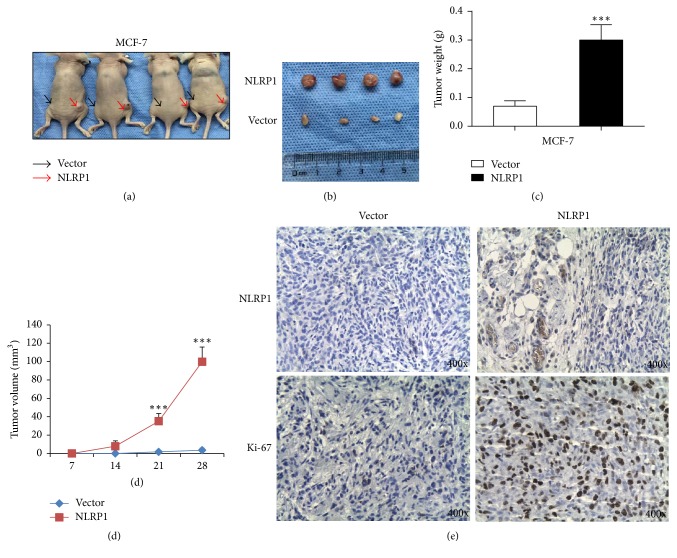
NLRP1 promoted the tumorigenicity of breast cancer* in vivo*. (a) Tumors were derived from vector (black arrows) and NLRP1-expressing (red arrows) MCF-7 cells in nude mice. (b) Tumors extracted from vector and NLRP1-expressing MCF-7 cells in nude mice. (c) Tumors weight histogram of NLRP1-expressing tumor in nude mice compared to the vector transfected tumors. ^*∗∗∗*^*p* < 0.001. (d) Growth curve for NLRP1-expressing tumor in a nude mice compared to vector transfected cells. ^*∗∗∗*^*p* < 0.001. (e) Immunohistochemistry of Ki-67 and NLRP1 protein levels. Original magnification, ×400.

To evaluate the tumor-promoting capacity of NLRP1* in vivo*, NLRP1 transfected or vector transfected MCF-7 cells were injected into nude mice. Thirty days after injection, tumors were excised. MCF-7 cells stably transfected with NLRP1 or empty vector formed tumors (Figures 4(a) and 4(b)). However, tumor volume and weight were greater in the animals injected with NLRP1 transfected MCF-7 cells (Figures 4(c) and 4(d)). Ki-67 level was also greater in NLRP1 transfected MCF-7 cells (Figure 4(e)).

## 4. Discussion

NLRP1 is highly expressed in leukemia patients and can form a large signal-induced multiprotein complex, which plays a crucial role in the development of leukemia [6]. Herein, we report that NLRP1 is highly expressed in breast cancer tissue. It is less frequently expressed in normal breast tissue. High levels of NLRP1 were associated with clinic-pathological features including lymph node metastasis, TNM stage, and Ki-67 level in breast cancer patients. An increasing number of studies have demonstrated the importance of EMT in breast tumor occurrence, development, and drug resistance [24–29]. We found that NLRP1 transfection promoted breast cancer cell line MCF-7 proliferation, migration, and invasion by possibly activating EMT and inflammasomes, mesenchymal markers vimentin, C-myc, MMP-9, and snail were upregulated, and then epithelial marker E-cadherin was downregulated. What is more, the key elements of inflammasomes also upregulated, such as IL-1*β*, IL-18, and ASC.

To date, the pattern of expression and function of NLRP1 in human breast tumor has not been elucidated. Based on our research, we favor the hypothesis that NLRP1 may play a critical role in the tumorigenesis proliferation, migration, and invasion of human breast tumor. This study is the first time to describe the detailed function in solid tumor and uncover the interaction between NLRP1 and EMT in breast cancer. But, in this study, we have not yet completed the detailed mechanism that NLRP1 promotes the malignant processes of breast cancer. Further study is also needed to elucidate the mechanisms of NLRP1 and EMT in breast cancer, such as Wnt/*β*-catenin signaling. Maybe just like NLRP3, we speculate that NLRP1 becomes activated in tumor microenvironment by inflammatory factor, endogenous ATP, cell damage, HMGB1, or potassium efflux [16, 17, 30–33]. In the future, we will set about doing more investigations in the mechanism.

## Figures and Tables

**Table 1:** Expression of NLRP1 in primary breast tumors and in adjacent tissues.

Samples	NLRP1 expression		*p* value
	Low	High	
BrCa (*n* = 72)	12 (17%)	60 (83%)	*p* < 0.001
BA (*n* = 36)	26 (72%)	10 (28%)	

BrCa, breast cancer; BA, breast cancer adjacent tissues.

**Table 2:** Association of NLRP1 expression and clinic-pathological parameters of 72 breast cancer patients.

Characteristics	*n*	NLRP1 expression		*p* value
		Low expression	High expression	
*Total*	*72*	*12*	*60*	
Age (years)				0.343
<55	36	4	32	
≥55	36	8	28	
Tumor size (cm)				0.197
≤2.5	24	6	18	
>2.5	48	6	42	
Lymph node metastasis				0.003
No	30	10	20	
Yes	42	2	40	
Histological grade				1
I/II	37	6	31	
III	35	6	29	
TNM stage				0.003
I/II	20	8	12	
III/IV	52	4	48	
ER				0.117
Negative	32	8	24	
Positive	40	4	36	
PR				1
Negative	34	6	28	
Positive	38	6	32	
HER2				0.744
Negative	22	3	19	
Positive	50	9	41	
P53				0.43
Negative	36	4	32	
Positive	36	8	28	
Ki-67				<0.001
Negative	12	10	2	
Positive	60	2	58	

Estrogen receptor (ER); progesterone receptor (PR).
